# Antiretroviral drug supply challenges in the era of scaling up ART in Malawi

**DOI:** 10.1186/1758-2652-14-S1-S4

**Published:** 2011-07-06

**Authors:** Erik J Schouten, Andreas Jahn, Anne Ben-Smith, Simon D Makombe, Anthony D Harries, Francis Aboagye-Nyame, Frank Chimbwandira

**Affiliations:** 1 Management Sciences for Health, Lilongwe, Malawi; 2 CMED / HIV and AIDS Department, Ministry of Health, Lilongwe, Malawi; 3 ITECH, Malawi and University of Washington, Seattle, USA; 4 Maame Akua, Lilongwe, Malawi; 5 HIV and AIDS department, Ministry of Health, Lilongwe, Malawi; 6 International Union Against Tuberculosis and Lung Diseases, Paris, France; 7 London School of Hygiene and Tropical Medicine, London, UK; 8 Management Sciences for Health, Arlington, US

## Abstract

The number of people receiving antiretroviral treatment (ART) has increased considerably in recent years and is expected to continue to grow in the coming years. A major challenge is to maintain uninterrupted supplies of antiretroviral (ARV) drugs and prevent stock outs. This article discusses issues around the management of ARVs and prevention of stock outs in Malawi, a low-income country with a high HIV/AIDS burden, and a weak procurement and supply chain management system. This system for ARVs, paid for by the Global Fund to Fight AIDS, Tuberculosis and Malaria, and bypassing the government Central Medical Stores, is in place, using the United Nations Children’s Fund’s (UNICEF’s) procurement services. The system, managed by a handful of people who spend limited time on supply management, is characterized by a centrally coordinated quantification based on verified data from all national ART clinics, parallel procurement through UNICEF, and direct distribution to ART clinics. The model worked well in the first years of the ART programme with a single first-line ARV regimen, but with more regimens becoming available (e.g., alternative first-line, second-line and paediatric regimens), it has become more difficult to administer. Managing supplies through a parallel system has the advantage that weaknesses in the national system have limited influence on the ARV procurement and supply chain management system. However, as the current system operates without a central warehouse and national buffer stock capacity, it diminishes the ability to prevent ARV stock outs. The process of ordering ARVs, from the time that estimates are made to the arrival of supplies in health facilities, takes approximately one year. Addressing the challenges involved in maintaining ARVs through an efficient procurement and supply chain management system that prevents ARV stock outs through the establishment of a dedicated procurement team, a central warehouse and/or national buffer stock is a priority.

## Background

The number of people receiving antiretroviral treatment (ART) has increased considerably in recent years to reach approximately 5.25 million by the end of 2009 [1]. The majority of patients on ART (3.9 million) live in sub-Saharan Africa. Despite the impressive growth, ART coverage of the population in need, calculated on the basis of the 2010 World Health Organization (WHO) guidelines [2], is 37% for adults and 28% for children, and in eastern and southern Africa, 41% for adults and 32% for children [1]. With an unabated growth of ART cohorts managed at an ever-increasing number of facilities, procurement and supply chain management (PSM) systems for HIV/AIDS medicines in resource-constrained countries are facing unprecedented challenges, and ruptures of the PSM systems are becoming a growing concern.

A WHO survey in 2009 revealed that 36 (38%) out of 94 reporting countries had documented at least one stock out of antiretroviral (ARV) drugs in health facilities [1]. Interrupted supply of ARVs puts individual patients at risk of disease progression and death [3], jeopardizes public health due to development of ARV drug resistance, hampers progress towards universal access, and diminishes the credibility of ART programmes in the eyes of patients, the community and healthcare providers. An increase and spread of HIV drug resistance will necessitate a change of first-line ARV regimens, and these are without exception more expensive and increase the costs of national ART programmes. In Malawi, the number of people on ART is expected to double, from 250,000 in December 2010 to more than 500,000 by June 2015, and the number of ART facilities is expected to increase from 400 to more than 700 [4].

This article will discuss operational challenges and operational research directions around the management and prevention of stock outs of ARVs in Malawi, a low-income country in southern Africa with a weak PSM and health system.

## ARV drug supply management system in Malawi

In many low- and middle- income countries, the capacity of the PSM systems has always been weak [5]. The management of supplies for what is essentially a chronic disease needing lifelong therapy is becoming increasingly difficult with further scale up of HIV care and treatment.

### Responsibility for procurement and distribution

During the design of the plan for the national roll out of the ART programme in Malawi, the weakness of the PSM system was well understood, and the challenge for the HIV programme managers was to establish a PSM system for ARVs that performed better than the general existing supply management systems. The debate about whether to improve the national PSM system as part of the HIV programme design or to develop a parallel PSM system for ARVs was overtaken by events because in 2002, a World Bank mission advised that the government Central Medical Stores should not be used for the procurement of HIV and AIDS supplies with funding from the Global Fund to Fight AIDS, Tuberculosis and Malaria (the Global Fund) [6]. The United Nations Children’s Fund (UNICEF) was proposed instead, and UNICEF, through its supply division in Copenhagen, Denmark, has been procuring and distributing ARVs in Malawi since 2004.

### Public health approach

Malawi’s Ministry of Health worked together with partners, including UNICEF, to develop the PSM system for ARVs that has been in place since 2004. The system has functioned relatively well and there have been no major stock outs of ARVs in Malawi, unlike many other medicines and medical supplies. It was clear from the onset of the national ART programme that it could only be successful if it was based on the realities in the health sector, in other words, by following a public health approach, i.e., a simplified, standardized and structured approach [7].

The first two years of experience have been reported earlier [8] and, here, we briefly describe the model.

### National, simple and standardized

At the outset in 2004, the ART programme focused on the roll out of one first-line ART regimen using a fixed-dose generic combination of stavudine, lamivudine and nevirapine. This regimen was the only one available in all but a few health facilities in the country providing ART services. Two alternative first-line ARV regimens (in case of toxicity) and a second-line ARV regimen (in case of treatment failure) were available in only four ART clinics in the country.

ARVs were distributed using a pre-packed kit system with “starter packs” and “continuation packs”, which simplified the supply management [8]. Using this approach, the ART programme was scaled up quickly and without any drug stock outs in its first four years [7].

### A “push system” and “ceilings” for the number of new patients starting ART

To ensure some control on rational drug forecasting for new patients, ART sites were given a ceiling of the maximum number of patients they were allowed to start each month on treatment. These ceilings were established on the basis of the number of inpatient beds, the population served, HIV prevalence and tuberculosis case burden (serving as a proxy for HIV/AIDS burden, given the strong association between the two diseases). The ceilings could be (and were frequently) adapted to cater for a higher demand and available capacity to provide ART, but only after the additional drug supplies were in place. These ceilings allowed easy calculations to be made on the number of “starter packs” needed for each site.

### Quarterly monitoring of patient outcomes and ARV drug stocks

At the end of every quarter, cumulative treatment outcomes of all patients started on ART at each site and the number of patients retained alive and on treatment were recorded. This latter count (patients alive and on treatment) captured the number needing continuation therapy, which needed to be added to the number of new patients starting treatment. ARV stocks were also counted in the pharmacies, and these data allowed a system of rational drug forecasting to be put in place to calculate the number of “continuation packs” needed for each site.

### Using a parallel procurement and distribution system for ART

The PSM system for ARVs was set up in parallel to the government’s PSM system run through Central Medical Stores. It was, and still is, characterized by a centrally coordinated quantification on the basis of verified data from all ART clinics in the country, procurement through UNICEF’s supply division and direct distribution of drugs to ART clinics, bypassing Central Medical Stores. Distribution of a six-month supply of ARVs to ART clinics was planned to take place when remaining drug stocks were estimated to be equivalent to two months of consumption.

## Operational challenges and proposed solutions or research areas

As the national ART programme has grown, it has become clear that the ARV PSM system based on the tenets we have outlined has become increasingly difficult to manage. This is due to a number of reasons, such as increasing numbers of people on ART, increasing numbers of sites providing ART, and a greater diversity of different ARV regimens. We will describe the challenges faced since the early years of the model and possible solutions or research needed to strengthen the system and avoid ARV stock outs.

### ARV drug regimes

By the end of December 2010, 91% of patients were still on the standard first-line regimen [9]. However, the number of different ARV formulations available to primary level ART sites has increased over the years to nine, and in the whole programme to 19 (11 adult and eight paediatric). Moreover, tertiary ART clinics and non-governmental organizations have been requesting an increase in the number of formulations to provide more individualized care; some specialized private and non-governmental ART clinics use a larger number of ARV formulations.

It is clear that in a growing and maturing ART programme, the very limited number of ARVs used during the first years of the national programme cannot be maintained and a balance needs to be found between improving individual care and maintaining a well-functioning PSM system. To better inform this debate and national policy, cost-effectiveness studies could be carried out to compare sites using many different ARV formulations with the national programme. Such research would also be useful as a guide to other countries that may be struggling with this balance.

### Patient ceilings and forecasting

The system of patient ceilings helped to prevent stock outs in the first years of the programme. However, as the numbers of people on ART increased, the bulk of the ARV consumption shifted to patients already on ART and away from patients who were newly starting treatment. The system of ceilings therefore became superfluous. Programme managers also became more experienced in more accurately forecasting the number of people on ART and ARV supplies needed. However, the number of people shifting to alternative first-line ART regimens (for reasons of drug toxicity) and second-line ART (for reasons of treatment failure) has been more difficult to predict. This led at one time to over-supply of second-line ARVs and contributed to shortages and even site stock outs of alternative first-line ARVs, although the main factor for the stock outs were bottlenecks in the Global Fund grant disbursement.

**Figure 1:**
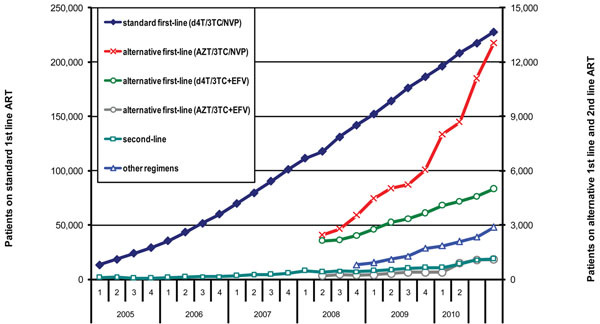
Number of patients on standard first-line (d4T/3TC/NVP), alternative first-line (AZT/3TC/NVP), alternative first-line (d4T/3TV/EFV), alternative first-line (AZT/3TC/EFV), second-line regimen and other regimens between 2005 and 2010 in the national ART programme in Malawi.

These shortages have influenced the erratic growth of the number of people on these regimens (Figure 1) and made forecasting of supplies needed more complicated. A potential area for operational research could be, therefore, the development and testing of models to predict the proportion of patients on treatment who need ARV substitution because of toxicity or switching due to treatment failure to help improve forecasting of second-line and alternative ARV consumption.

### Procurement processes

The traditional PSM system of providing health facilities with a consignment of supplies to ensure that stock positions in health facilities are maintained within the set range of “minimum-maximum stock position”, based on an average monthly consumption calculated using past consumption, does not work for ARVs as the patient burden grows and the consumption continually increases. The Global Fund requires that all ARVs are accounted for, and thus Malawi set up a monitoring and evaluation system in which patient data are audited and physical stock checks are performed by a supervision team visiting all ART clinics on a quarterly basis [10].

UNICEF is requested by the Ministry of Health to prepare “cost estimates” for the required supplies. Once these have been received, the ministry verifies the “cost estimates” and contacts the Principal Recipient of the Global Fund grant, the National AIDS Commission, to prepare a Disbursement Request to the Global Fund. The Local Funding Agency performs some checks to verify if the request is correct, following which the Disbursement Request is sent to the Global Fund for final approval. Once this has been obtained, the monies are transferred to UNICEF’s Supply Division and “purchase orders” are issued.

**Figure 2:**
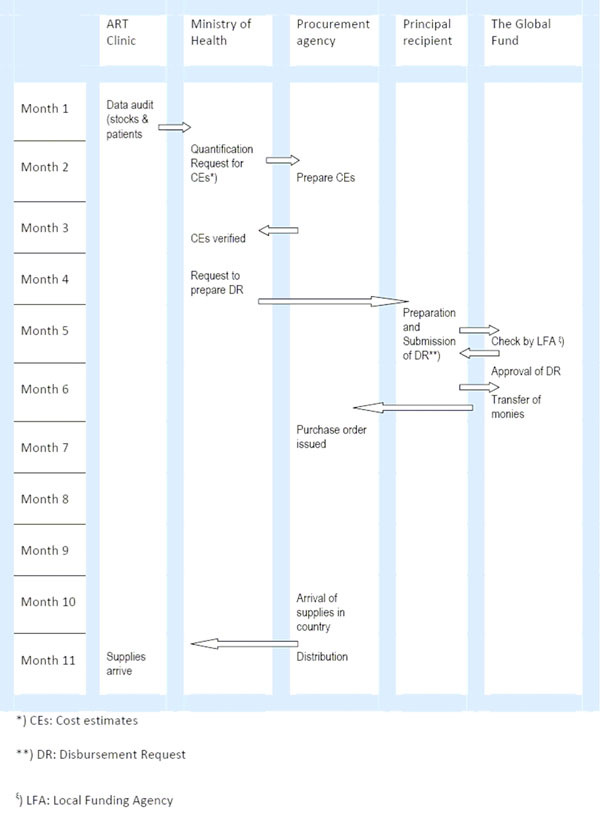
Outline of the steps involved in procurement of ART drugs, from quantification by the Ministry of Health to the receipt of supplies at the health facilities.

ARV shipments usually arrive some four months after the purchase orders have been placed, depending on the manufacturers’ production capacity and air transport to Malawi. Supplies are then distributed by UNICEF to health facilities within a month after arrival in country. The process from quantification to receipt of supplies at the health facilities is complex, lengthy and involves multiple players (Figure 2). Orders of ARVs that are made on the basis of stock positions and the number of people on ART at the end of June 2011 will not arrive in health facilities before May 2012, almost one year later.

The long procurement process and increasing quantity of ARVs pose major challenges to prevent stock outs. First, predictions have to be made for one year ahead. While a degree of confidence is applied around some of the estimates for each site, with an obvious tendency to over-order instead of under-order, it may be necessary to increase the in-country stocks of ARVs to cover the eventuality of potential delays in the process.

Second, the current parallel supply management system operates without a central warehouse and a national buffer stock, which diminishes the in-country capacity to prevent stock-outs of ARVs. The buffer stock, or safety stock, is the minimum stock that is kept on hand to protect against stock out. If there is no safety stock, stock outs will occur when deliveries are delayed or when there is an unexpected increase in demand [11]. As the ART programme has grown, the quantity of ARVs stored in health facilities has increased, sometimes beyond capacity. There is therefore a pressing need to have a central warehouse facility. This would need to be properly managed to ensure that stored drugs do not pass their expiry dates.

Third, if a warehouse was set up, the option of having a national buffer stock would become feasible. Experience in Malawi has shown that once the national stock position (which reflects all stock now available and soon to be available, including stock on hand and outstanding orders [11]) drops below two months of consumption (based on drug stock data collected from all national ARV sites), the prevention of stock outs becomes very difficult, and a lot of time and energy is put into re distribution of supplies. We believe that the establishment of a national buffer stock that would maintain the national stock position at six months’ consumption is a priority.

Such a proposal has been put forward to the Global Fund, who has agreed to fund it. Fourth, plans have been developed to increase the frequency of distribution to health facilities from six monthly to three monthly to reduce the average quantity of ARVs held in health facilities; however, this cannot happen until a central warehouse and a national buffer stock are established. More frequent distributions will not only reduce the pressure on storage space in health facilities, but also facilitate the response to unexpected changes in demand as redistributions take place between health facilities.

### Institutionalization of the PSM system for ARVs

The work of keeping the PSM system for ARVs on track is currently in the hands of very few people in the Department of HIV and AIDS in the Malawi Ministry of Health (monitoring and evaluation officer, HIV care and treatment officers) and the National AIDS Commission (director of finance, chief procurement officer), who spend only part of their time on supply management. The value of the ARVs for the national ART programme will increase from US$60 million per year in 2011 to $100 million in 2015. The value of these supplies is high in comparison with the government’s contribution to health, which was estimated at US$65 million in 2006 [12].

There is therefore an urgent need to have an appropriately qualified team, including staff from the Central Medical Stores, dedicated to procurement and supply management for ARVs and other HIV commodities, possibly combined with the procurement of other supplies procured with Global Fund monies. This team would need to take full responsibility, and be accountable, for procurement and distribution. Once such a team was in place, it would be important to carry out operational research to document whether this led to a reduction in the quantity of ARVs expiring and the frequency of (near) stock outs of ARVs in health facilities.

### Dependency on a single donor for drug ordering and procurement

Adequate funding for ARVs is a *conditio sine qua non*. Paying for ARVs by patients is associated with higher loss to follow up [13] and poorer treatment outcomes, and WHO advises therefore that ART should be free of charge in low- and middle-income countries [14]. Countries with a small economy, like Malawi, are not able to contribute significantly to the procurement of ARV supplies. Malawi’s gross domestic product is US$318 (per capita) [15] and the government contribution to health was approximately US$65 million ($5.4 per capita) in 2005-2006. This is insufficient to procure a considerable proportion of the annual need of ARVs.

The vast majority of ARVs are procured with funds from the Global Fund and the lack of existing alternative funding sources makes the programme very susceptible to any bottlenecks in grant disbursement. Unforeseen delays in the PSM system over the past two years has led to periods of very low ARV stock levels, and even a few episodes of stock outs in a small number of health facilities. Most of these episodes were related to the late disbursement of funds, and virtually all steps prior to the disbursement of funds (see Figure 2) have contributed at least once to these delays. As a first step, it is important for all stakeholders to agree on what are the key steps and bottlenecks required to move a drug order through to drug distribution in the field, and then to find potential solutions to these barriers. Time-related targets should be set for each step, and then operational research should be conducted every six months to determine if targets were reached and reasons for not reaching targets.

Another recent and interesting development is the establishment by the UN Foundation of the “Pledge Guarantee for Health” to help grant recipients to accelerate health commodity procurement. The pledge will support a bank to issue a letter of credit to a supplier on behalf of a grant recipient, which in principle should facilitate and speed up the procurement of supplies [16]. This mechanism has yet to be used.

### Changes in recommended ART regimens

WHO launched revised guidelines for prevention of mother to child transmission and ART in 2010 [2,17]. These guidelines advise changing the eligibility criteria to start ART and propose a move away from stavudine-containing ART regimens to regimens with less toxicity. The number of people eligible for ART increases by an estimated 45%, and the recommended ARV regimens are at least twice as expensive in comparison with the stavudine-containing regimens. Any associated changes in national recommendations as a result will require careful monitoring to ensure that past regimens will still be used until the stock is finished and before the expiry date and that the new regimen will be available in sufficient quantities to cover the needs (with progressive inclusion on new regimens). Malawi will then move on changing the ARV regimens.

This process, and the scale of the change, has no precedent. It will require a phasing out of the old first-line regimen so as not to waste drugs and introducing the new regimen with all that this entails for patient and community understanding and healthcare delivery. Operational research to carefully document such processes will be essential so that lessons can be learnt for other countries embarking on the same course and for additional regimen changes in the future.

## Conclusions

### Lessons learnt

From the start of ART scale up six years ago, important lessons have been learnt. Table 1 gives an overview of the challenges, proposed way forward and the ways in which operational research can guide future policies. First, an increase in the number of different ARV formulations has led to a more complicated PSM system and has increased the risks of stock outs. The national ART programme therefore needs to maintain a careful balance between the optimization of individual care and a limited number of standard regimens to simplify and/or facilitate the supply management system.

**Table 1:** An overview of the challenges involved in ART drug supply management, proposed way forward and the ways in which operational research can guide future policie

Challenges	Way forward	Proposed operational research
Increased number of different ARV formulations complicates the supply management system and increases the risk of stock outs	Keep the number of different ARV formulations as small as possible while maintaining adequate treatment options for the vast majority of patients	Cost-effectiveness studies comparing ART sites with large number of different ARVs and the national programme
Correctly forecasting the proportion of patients in need of changing of ART regimen	Ensure that adequate alternative and second-line ARVs are available	Develop and test models that predict proportion of patients in need of substitution or switching ARV regimens Compare forecasting system based on patient numbers (ART clinic data) and ARV consumption (pharmacy data) in ART sites to decide which is the best predictor
National stock positions that have decreased to two months of consumption lead to near stock outs in some ART clinics	Increase national stock positions to a minimum of six months’ consumption	Document the number of (near) stock outs after the establishment of the national buffer stock
Storage capacity in health facilities is limited and undermines the quality of supply management	Increase frequency of deliveries to ART clinics from six monthly to three monthly Establish a national buffer stock	Document the number of (near) stock outs and the quantity of ARVs that expires
ARV supply management is managed by a few individuals in the Ministry of Health	Hire/establish a dedicated full-time team for supply management for HIV commodities	Document the number of (near) stock outs
Disbursement of grants is often interrupted	Increase national capacity to manage ARV supplies including reporting to donors Establishment of a fund that will lend monies to HIV programmes as suggested by the United Nations Foundation (‘Pledge Guarantee for Health’)	Document the timely release of funding by the main donor (The Global Fund)

Second, minimum national stock positions of ARVs of two months’ consumption often lead to very low stock positions in individual ART sites and additional work due to redistribution and repositioning of ARVs. National minimum stock positions should therefore increase. As the storage capacity for medicines at health facilities is limited and calculated based on stock positions of three months’ consumption, the most feasible option is therefore to increase the national storage capacity through the establishment of a central stock. Increasing the national stock positions in country to six months of consumption will allow supplies to arrive four months later than planned without causing supply interruption. Increasing the national stock position further would increase the robustness of the supply system. However, increasing the minimum national stock position from two months to six months of consumption would cost US$18 million on the basis of the non-stavudine-based first-line ARV regimen.

Third, increasing the frequency of deliveries from the central warehouse to ART sites would further decrease pressure on storage capacity in ART sites. These two measures would not only improve supply management of ARVs, but would also mitigate the effects of delays in grant disbursement.

Fourth, the supply management of ARVs is carried out by a few individuals who are not fully dedicated to this activity. Given the increasing volumes of ARVs and other HIV commodities, a dedicated full-time team of staff experienced in supply management is needed to further develop the ARV PSM system and ensure its institutionalization.

Fifth, disbursement of grants needs to be streamlined.

## Competing interests

No competing interests are declared.

## Authors’ contributions

EJS, AJ and ABS wrote the first draft of the manuscript. All authors were involved in the revision of the manuscript and approved the document for publication.
